# Comparison of acellular dermal matrix allograft (ADMA) and a subepithelial connective tissue graft (SCTG) for the treatment of gingival recession

**DOI:** 10.34172/japid.2020.004

**Published:** 2020-04-21

**Authors:** Niloofar Jenabian, Mohadese Yazdanpanahbahabadi, Parya Haghpanah Aski, Ali Bijani

**Affiliations:** ^1^Department of Periodontics, Dental School, Babol University of Medical Sciences, Babol, Iran; ^2^Department of Non-communicable Pediatric Diseases Research Center, School of Dentistry, Babol University of Medical Sciences, Babol, Iran

**Keywords:** Allograft, Autologous, Gingival recession, Tooth root, Transplantation, Transplants

## Abstract

**Background:**

This study aimed to evaluate the effect of acellular dermal matrix allograft (ADMA) for the treatment of gingival recession as a substitute for subepithelial connective tissue graft (SCTG).

**Methods:**

In this controlled clinical trial, 18 teeth were selected in nine subjects with bilateral gingival recession. One side was treated with SCTG and a coronally displaced flap as the control group, and the other side was treated with ADMA and a coronally displaced flap as the test group. Probing pocket depth (PPD), clinical attachment level, vertical recession depth, recession width, gingival thickness, keratinized tissue width, and the root coverage percentage were measured before the surgery and at 1-, 3-, and 6-month postoperative intervals. The healing index, pain index, and patient satisfaction were also investigated. The data were analyzed with a general linear model (GLM) repeated measures and paired t-test.

**Results:**

All the parameters improved except for PPD; however, a comparison between the groups did not reveal statistically significant differences. Only root coverage percentage and pain index were significantly lower in the test group. The average percentage of root coverage in the control and test groups were 82.01±16.62% and 64.44±9.4%, respectively.

**Conclusion:**

Both methods resulted in improvements in the clinical results. However, the use of the ADMA led to less pain and root coverage in comparison with the SCTG method.

## Introduction

“Gingival margin situated in apical of the cementoenamel junction” has been considered as the gingival recession, which could lead to the exposure of the root surface and loss of the attached gingiva.^1^ In more than 50% of people, one or more gingival recessions with <1 mm height have been observed.^2^ The gingival recession might cause an increase in tooth sensitivity,^3^ pain, an unaesthetic appearance of the gingiva, and loss of periodontal attachment;^4^ it may also make dental healthcare difficult.^5^ Among all the factors which cause gingival recession, the most common reason is the traumatic and abrasive use of the toothbrush, which can involve the buccal surfaces of the teeth;^6^ also, gingival recession could happen as a result of inflammatory conditions, and it generally presents in patients with periodontitis.^7^

It seems that the most important factor which increases the risk of the gingival recession is the thin gingival biotype covering the thin marginal tissue.^8^

Various methods have been applied to cover the roots, including the autogenous free gingival graft,^9,10^ the autogenous connective tissue graft,^11^ pedicle autografts (laterally^12^ and coronally^13^ positioned flaps), subepithelial connective tissue grafts (SCTG),^14^ tissue engineering techniques^15^ (such as acellular dermal matrix allograft), and the use of biological mediators to prevent the progression of gingival recession, facilitate the plaque control, protect the keratinized gingiva, reduce the high frenum activity, and reduce tooth hypersensitivity.^16^ SCTG has the advantages of both free gingival autografts and pedicle grafts. The high survival rate of the subepithelial connective tissue graft is attributed to the existence of two blood sources, namely the facial gingival tissue flap and the exposed bed of the root zone environment. Although the subepithelial connective tissue graft is the gold standard,^17,18^ pain, the patient’s inconvenience, the sufficiency of the donor area, bleeding of the harvesting region, and a need for a second surgery are the complications of this method. Therefore, the acellular dermal matrix allograft has been widely applied as a substitute for autogenous tissue grafts in mucogingival surgeries.^19-21^ This allograft is made of human corpse skin through decellularization, freezing, and drying processes to prevent inflammation or rejection of the transplant. However, the essential factors of the tissue structure, such as collagen, elastin, proteoglycans, and vascular channels, are maintained in the process.^22-24^ According to the studies, due to the presence of vascular channels in the region, ADMA would be integrated with the host tissue and preserve its structural integrity.^25-30^

Many studies concluded that the application of ADMA is comparable to subepithelial connective tissue graft. The mean root coverage of these two methods did not show clinically significant differences.^30-33^

In this regard, it seems that this method could be an appropriate alternative to connective tissue grafts, especially in young and older people, and also those who are not systematically fit for intensive surgeries.

Recently, this allograft has been produced in Iran by the Tissue Regeneration Corporation (TRC) under the proprietary name Cenoderm. It would be more appropriate for both the patient and clinician due to its availability and price because it is manufactured in Iran. The present study was conducted to clinically compare the acellular dermal matrix allograft (Cenoderm) and subepithelial connective tissue graft.

## Methods

Eighteen teeth were selected in nine subjects with bilateral gingival recession for this randomized, double-blind controlled, split-mouth study. The inclusion criteria were as follows: (1) ≥18 years of age, (2) ability to maintain proper oral hygiene (O’Leary plaque score^34^ ≤ 20%), (3) all recessions in either Miller I or II category, (4) bilateral isolated buccal gingival recession with a depth of at least 2 mm from the cementoenamel junction (CEJ) in the anterior and premolar teeth, (5) no filling and bleeding upon probing in the selected teeth (6), and adequate vestibular depth. The exclusion criteria were defined as follows: (1) pregnancy, (2) confounding medications interfering with wound healing (e.g., anti-neoplastic agents and corticosteroids), (3) cigarette smoking, (4) traumatic methods of brushing and application of abrasive toothpaste, (5) a history of periodontal surgery in the past two years, (6) wearing removable prostheses or orthodontic appliances in the designated area, (7) long-term (i.e., >2 weeks) use of antibiotics in the last three months, and (8) known allergies to the materials used during periodontal surgery.

This study was reviewed and approved by the Ethics Committee of Babol University of Medical Sciences and registered at http://www.irct.ir (registration number: IRCT201305201760N23; registration date: 2013-08-23). All the enrolled patients were provided with an oral explanation of the study, and written informed consent was obtained.

### Randomization and blinding

The patients were treated either with subepithelial connective tissue graft and a coronally positioned flap or an acellular dermal matrix allograft with a coronally positioned flap.

A 4-interval permuted block method was used to assign the patients to receive any type of intervention on the right side (i.e., arbitrarily selected). The assignments were sent in closed envelopes to the clinician in charge (PH), who was unaware of the assignment codes. Further measurements of periodontal indices were performed by another clinician (NJ), who was also blinded to the study arms. All the surgeries were performed by the same clinician, and the measurements and randomization were made by another clinician.

### Study design

Phase I periodontal therapy was performed for all the patients. All the procedures were performed by the same person (PH). After adequate and profound local anesthesia (i.e., infiltration method) with 2% lidocaine containing epinephrine at a concentration of 1:80000, all the exposed roots were carefully prepared by scaling and root planing.

**Figure 1:**
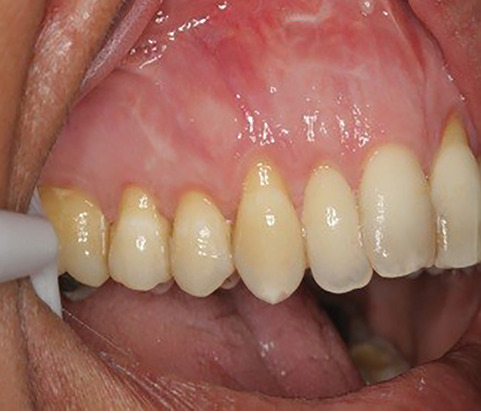
SCTG site before surgery.

**Figure 2:**
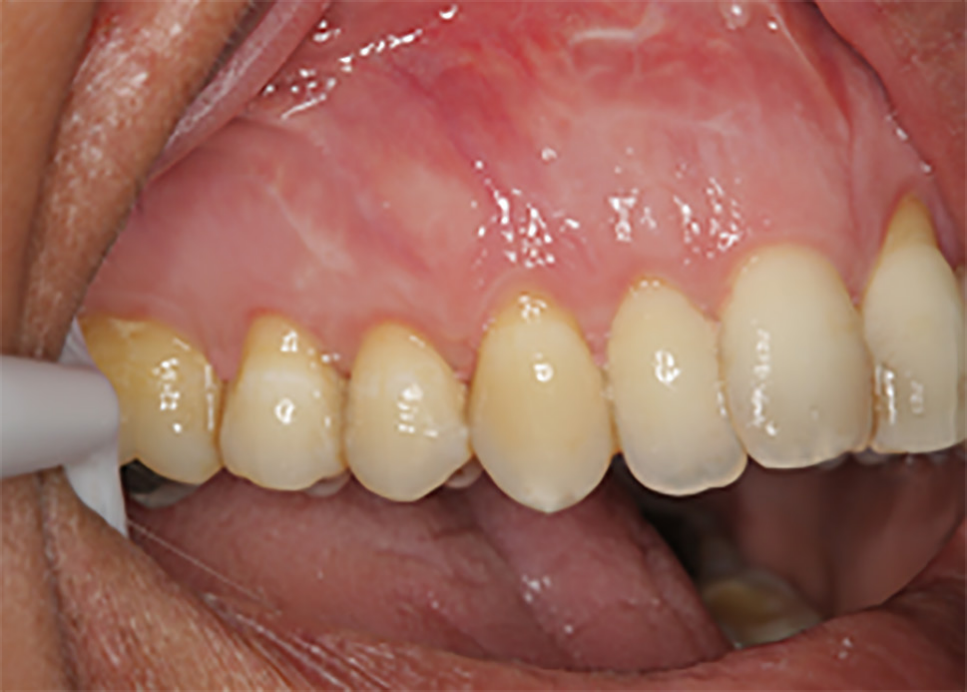
SCTG site 6 months after surgery.

**Figure 3:**
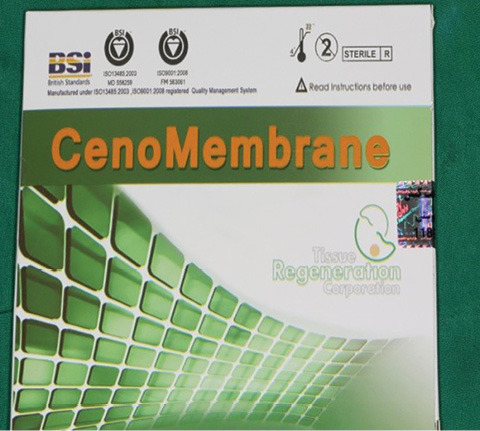
Cenoderm.

**Figure 4:**
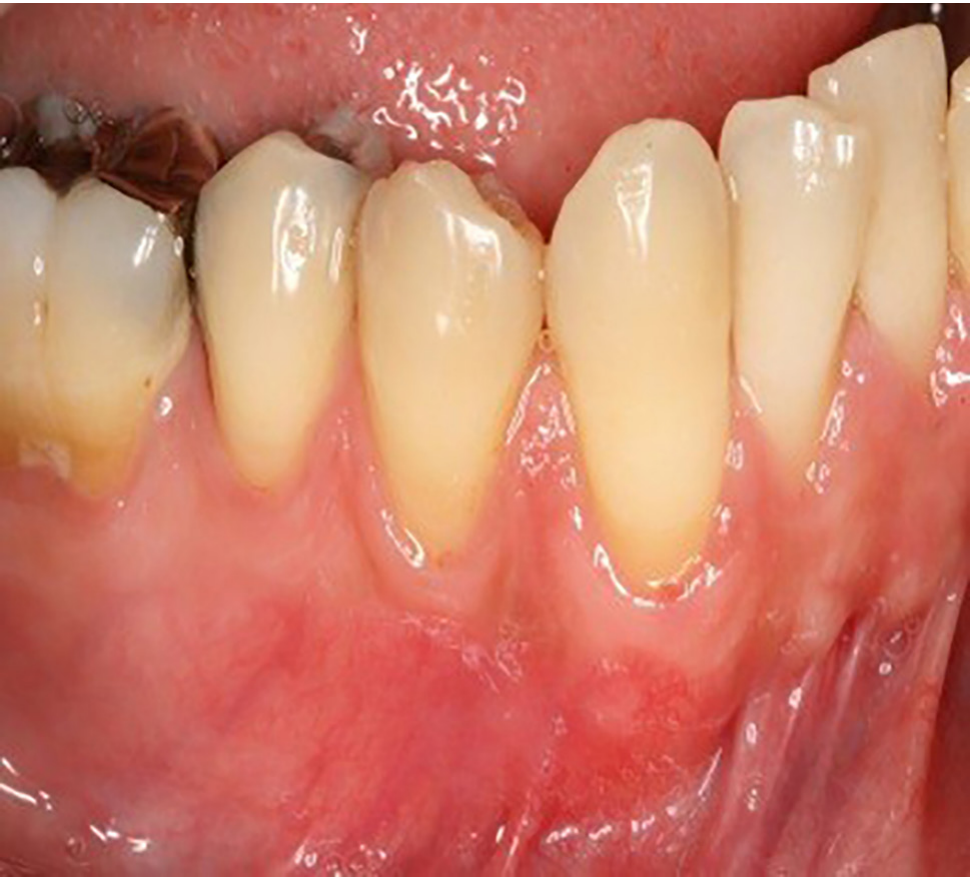
Cenoderm site before surgery.

**Figure 5:**
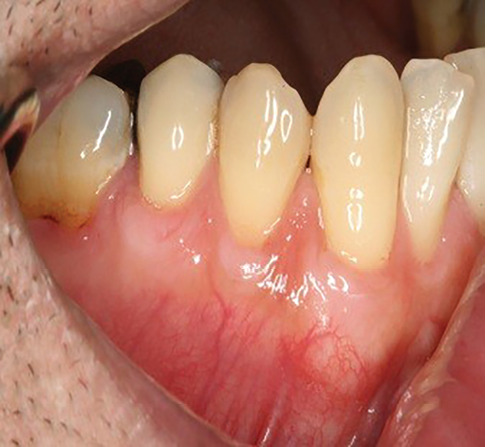
Cenoderm site- 6 months after surgery.

The sulcular incision was made at the recipient site. The flap was beveled in the interdental papilla region adjacent to the tooth with the exposed root. A partial-thickness flap was raised beyond the mucogingival junction. The mesiodistal width of the incision was extended to the line angle of the adjacent teeth mesially and distally. Mesial and distal vertical releasing incisions were also made. The required graft width was measured using a periodontal probe. A connective tissue graft was prepared from the palate, measuring 1–1.5 mm in thickness and fixed at the recipient site with a 4-0 bio-absorbable polyglycolic suture. Then, a coronally repositioned flap was relocated to cover the graft and stabilized in the site with a sling suture (Figures 1 and 2). A 10×20-mm and 0.6–0.9-mm-thick graft was prepared from acellular dermal matrix graft [Cenoderm, Tissue Regeneration Corporation (TRC), Iran] (Figure 3) according to the manufacturer’s instructions. The graft was placed at the recipient site from its porous surface from the CEJ to 2–3 mm beyond the bony margin of dehiscence and fixed at the recipient site with a 4-0 bio-absorbable polyglycolic suture. Then, a coronally repositioned flap was relocated to cover the graft and stabilized with a sling suture (Figures 4 and 5).

A non-eugenol periodontal dressing was placed on the donor site, which was removed along with the sutures approximately two weeks later. All the patients were instructed to rinse with %0.12 chlorhexidine twice a day for four weeks. A systemic antibiotic (penicillin VK, 500 mg, q.i.d.) and a pain killer (ibuprofen, 400 mg, q.i.d.) were prescribed for a week.

### Primary and secondary endpoints

Baseline measurements were recorded using a periodontal probe, and the records were rounded to the nearest 0.5 mm using a University of Michigan “O” probe with Williams marking. The recorded indices were as follows: keratinized tissue width (KTW, the distance from the free gingival margin to MGJ), clinical attachment level (CAL, the distance from the CEJ to the pocket floor), probing depth (PD, the distance from the free gingival margin to the pocket floor; the average of mesiobuccal, mid-buccal, and distobuccal measures was taken into account), recession depth (RD, the distance from the CEJ to the free gingival margin measured at mid-buccal area), recession width (RW, measured at one mm apical to the CEJ, in a mesiodistal direction), gingival thickness (GT, measured two mm apical to the free gingival margin by gently inserting the probe into the tissue on the buccal side) and esthetic index (E-VAS, using a visual analog scale from 0 to 10).^35^ All these measurements were repeated one, three and six months after the periodontal surgery. In addition, the healing index (Landry’s index) (Table 1)^36^ was recorded at baseline, and three days, one week, and one month later. All the patients reported their pain one, three, and seven days after the surgery using a visual analog scale (VAS, from 0 to 10) diagram.^35^ The root coverage index was obtained from the following equation: (Recession depth at baseline – Recession depth at the 6th month)/Recession depth at baseline) ×100. In all the sessions, clinical photographs were also taken.

**Table 1:** Healing index by Landry et al

**1. Very poor**	• Tissue color: ≥50% of the gingiva red • Response to palpation: bleeding • Granulation tissue: present • Incision margin: not epithelialized, with loss of epithelium beyond the incision margin • Suppuration present
**2. Poor**	• Tissue color: ≥50% of the gingiva red • Response to palpation: bleeding • Granulation tissue: present • Incision margin: not epithelialized, with the connective tissue exposed
**3. Good**	• Tissue color: ≥25% and <50% of the gingiva red • Response to palpation: no bleeding • Granulation tissue: none • Incision margin: no connective tissue exposed
**4. Very good**	• Tissue color: <25% of the gingiva red • Response to palpation: no bleeding • Granulation tissue: none • Incision margin: no connective tissue exposed
**5. Excellent**	• Tissue color: all the tissues pink • Response to palpation: no bleeding • Granulation tissue: none • Incision margin: no connective tissue exposed

### Study population

The subjects were recruited from patients referred to the Department of Periodontics, Dental School, Babol University of Medical Sciences, Babol, Iran. Patients with bilateral gingival recession were selected from enrolled patients and randomly recruited in the clinical trial.

### Statistical analysis

The sample size was estimated at nine subjects (n=9) to achieve an 80% power for a standardized difference of 1.8 between the two study arms, calculated for the primary endpoint (recession depth), using an Altman’s nomogram. Continuous data were expressed as means (±standard deviations). Kolmogorov-Smirnov test was used to assess the normal distribution of the data. The changing trend of each index within each study arm group was traced using GLM repeated measures (RM) statistics. Sphericity (one of the GLM-RM assumptions) was tested with Mauchly’s test, and the data were adjusted and reported with epsilon Greenhouse–Geisser correction in case of violation.

Furthermore, a Bonferroni test was used to evaluate differences between the groups. The Friedman test was applied to trace the changing trends of nonparametric data. Moreover, the mean difference in each non-normally distributed index was calculated from the beginning to the end of the study and compared with a paired t-test. A two-tailed α at P<0.05 was considered statistically significant.

## Results

### Baseline characteristics

Baseline indices of the patients at T_0_ were compared between the two groups to figure out any possible confounding effect due to the baseline differences. The t-test showed no significant difference in any of the indices (P<0.05).

### Primary and secondary endpoints (Overall group analysis)

**Figure 6:**
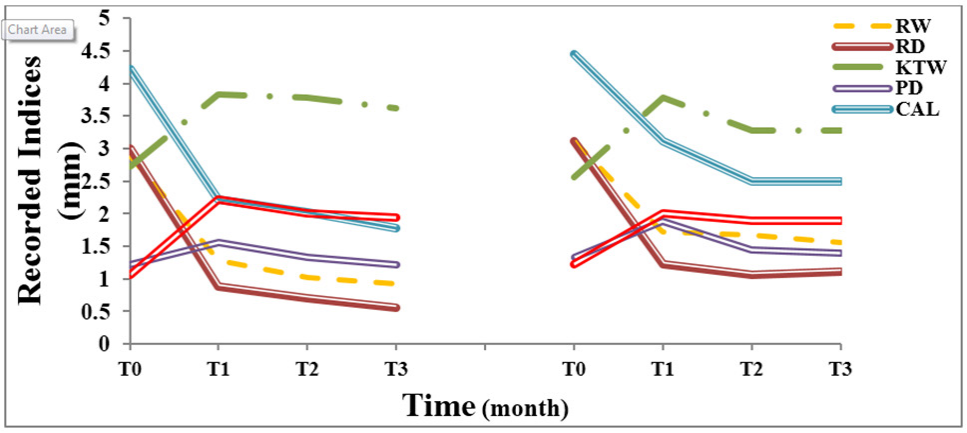
The left side displays SCTG data, and the right side displays ADMA data. RW: recession width; RD: recession depth; KTW: keratinized tissue width; PD: probing depth; CAL: clinical attachment level; GT: gingival thickness; T_0_: baseline; T_1_: first month; T_2_: third month; T3: sixth month).

**Figure 7:**
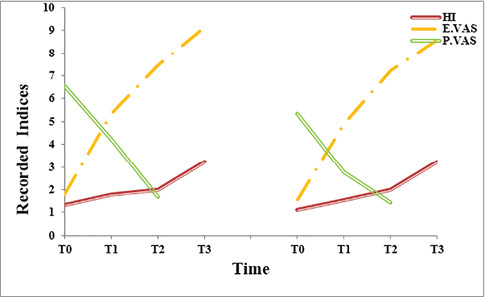
The left side displays SCTG data, and the right side displays ADMA data. HI: healing index (T_0_: baseline, T_1_: third day, T_2_: first week, T_3_: first month); E.VAS: esthetic visual analogue scale (T_0_: baseline, T_1_: first month, T_2_: third month, T_3_: sixth month); P.VAS: pain visual analogue scale (T_0_: first day, T_1_: third day, T_2_: seventh day).

Changing trends of measured indices were all statistically significant, as shown in Figures 6 and 7. Except for probing depth [X^2^(3)=10.05, P=0.02)], others improved, including recession depth (F_(1.94)_=84.15, P<0.001), recession width (F_(1.93)_=77.93, P<0.001), the width of keratinized gingiva (F_(1.66)_=37.84, P<0.001), clinical attachment level (F_(1.84)_= 45.04, P<0.001), pain (F_(2)_=83.78, P<0.001), thickness of keratinized gingiva [X^2^(3)=46.31, P<0.001)], healing index [X^2^(3)=47.76, P<0.001)], and esthetic [X^2^(3)=53.72, P<0.001)].

### Primary and secondary endpoints (Inter-group analyses)

In intergroup comparisons, patients in the SCTG group reported greater pain [(SCTG=4.19±0.18), ADMA=3.19±0.18, mean diff=0.96, P=0.001, power=0.95)] and displayed better root coverage [(SCTG= 82.01±16.62%), (ADMA=64.44±9.4%), (t(8)=3.43), (mean diff=5.43), P=0.009)]. Meanwhile, other indices were not in contrast with the study groups: esthetic improvement [(SCTG=7.33 (±1.11), (ADMA=7±0.87), (t(8)=2), (mean diff=0.33), P=0.08)], improved healing [(SCTG=1.88±0.35), (ADMA=2.1±0.6), (t(8)=1.5), (mean diff=0.22), P=0.17)], increased thickness of attached gingiva [(SCTG=0.89±0.33 mm), (ADMA=0.6±0.33 mm), (t(8)=1.51), (mean diff=0.22), P=0.17)], probing depth change [(SCTG=0±0.71 mm), (ADMA= -0.06±0.73 mm), t((8)= -0.19), (mean diff=0.05), P=0.86)], improved clinical attachment level [(SCTG=2.56±0.3 mm), (ADMA=3.14±0.3 mm), (mean diff= 0.58), P=0.19, power=0.25)], gained width of keratinized gingiva [(SCTG=3.48±0.68 mm), (ADMA=3.22±0.68 mm), (mean diff=0.26), P=0.79, power=0.06)], improved recession depth [(SCTG=1.29±0.29 mm), (ADMA=1.63±0.29 mm), (mean diff=0.34), P=0.42, power=0.12)] and improved recession depth [(SCTG=1.52±0.27 mm], (ADMA=2.02±0.28 mm), (mean diff=0.49), P= 0.23, power=0.40)] (Figure 6 and 7).

## Discussion

In this research, CAF + Cenoderm (test group) was compared with CAF + SCTG (control group) for the treatment of Miller class I/ll buccal gingival recession.

No significant difference was observed between the two groups in any of the parameters at baseline. The average percentage of root coverage in the Cenoderm group was significantly less than that in the SCTG group (64.44±9.4% and 82.01±16.62% in test and control groups, respectively), consistent with the findings reported by Novaes et al,^37^ Barros et al,^38^ Bouchard et al,^39^ and Agarwal et al,^40^ which were expected because the subepithelial connective tissue graft is a gold standard method.

In the present study, the average clinical attachment gain improved in both groups, with no significant difference between them, consistent with the result of the studies by Novaes et al,^37^ Haghighati et al,^41^ Hirsch et al,^42^ and Juluri et al.^43^

The changes in probing depth were not significant in the two groups, indicating that PPD did not change during the study, consistent with several previous studies^37,41,44,45^ but not all of them.^46^ These differences in PPD values could be attributed to the variations in the initial PPD and differences in examiners.

According to Gholami et al,^45^ the use of SCTG leads to more gains in the keratinized gingiva compared to ADMA. They believed that this is because ADMA needs more time for keratinization of the epithelial surface. However, a study by Novaes et al^37^ was consistent with this study, which showed an increase in the keratinized tissue width in both methods (SCTG and ADMA) after six months, with no significant difference between them.

The mean average of depth and width reduction of gingival loss was enhanced in both groups, with no significant difference between them, consistent with the results reported by Novaes et al,^37^ Rahmani et al,^44^ Gholami et al,^45^ and Shori et al.^46^

Despite the difference in the amount of coverage between the two groups, there was no difference between these two treatment methods in terms of the VAS index for esthetic.

An assessment of tissue repair was carried out by Landry index in each group, which showed an improvement, with no significant difference between the two groups.

The indices of healing, esthetic, and pain have not been investigated in other studies.

The gingival thickness, too, improved in both groups, with no significant difference between them. VAS for pain showed an apparent difference between the two groups. The pain was considerably less severe in the test group in comparison with the control group. These results were somehow predictable because the surgical site was more extensive with the SCTG technique compared to the ADMA technique. According to this study, patients used more analgesics for a longer duration.^47^ These results could be considered as one of the advantages of the ADMA method.

Furthermore, it is possible to treat a broader region in this technique in comparison with the other techniques, such as SCTG. The advantage of fewer complications of ADMA (Cenoderm) could be exploited in young people under orthodontic treatments and older people with systemic problems, especially those with bleeding disorders.

## Conclusion

According to the results of this study, both SCTG and Cenoderm resulted in improvements in the clinical outcomes. However, the use of Cenoderm led to less severe pain and less root coverage in comparison with the SCTG technique.

## Authors’ Contributions

NJ: Executor, MY: Executor, PHA: Executor and corresponding author, AB: Statistics Consultant. All authors have read and approved the final manuscript.

## Ethics Approval

The study protocol was approved by the Ethical Committee of Babol University of Medical Sciences and registered at http://www.irct.ir (registration number: IRCT201305201760N23; date registered: March 23, 2013).

## Competing of Interests

Yes- Cenoderm (Tissue Regeneration Corporation).
